# Development and Validation of an Enzyme-Linked Immunosorbent Assay for Measuring Factor XI in Intravenous Immunoglobulin Products by Mitigating Heterophilic Antibody Interference

**DOI:** 10.4014/jmb.2601.01027

**Published:** 2026-04-03

**Authors:** Yeon-Jung Kim, Hyewon Lee, Jeungwoon Hong, Yeeun Lee, Jae-Hwan Nam

**Affiliations:** 1 GC Biopharma Corp., Yongin-si 16924, Republic of Korea; 2 Department of Medical and Biological Sciences, The Catholic University of Korea, Bucheon 14662, Republic of Korea

**Keywords:** Enzyme-linked immunosorbent assay, Factor XI, Intravenous immunoglobulin

## Abstract

Thromboembolic events associated with intravenous immunoglobulin (IVIG) therapy have been linked to activated coagulation factor XI (FXIa). Therefore, regulatory authorities recommend monitoring FXI levels in IVIG products using quantitative assays, such as sandwich enzyme-linked immunosorbent assays (ELISAs). However, high concentrations of immunoglobulins in IVIG can cause heterophilic antibody interference, leading to false-positive FXI measurements. In this study, we aimed to develop and validate a sandwich ELISA capable of accurately measuring FXI while minimizing interference from high immunoglobin G (IgG) concentrations. We hypothesized that Purified human IgG (Fc) could serve as a heterophilic antibody blocker, thereby neutralizing non-specific interactions and improving assay reliability. The FXI measurements were compared with and without IgG blocker treatment across process intermediates and final IVIG products with varying IgG levels. False-positive signals were observed only in samples with high IgG concentrations and were eliminated after treatment with the IgG blocker. The modified ELISA demonstrated acceptable accuracy, precision, linearity, and quantification limits in accordance with the ICH Q2(R^2^) guidelines. This validated assay reliably quantified FXI regardless of IgG concentration, providing a robust platform for impurity assessment during IVIG manufacturing.

## Introduction

Intravenous immunoglobulin (IVIG) is used to treat primary immunodeficiency disorders, which are also known as congenital immunodeficiency. It comprises a mixture of antibodies extracted from the plasma of healthy donors. However, numerous cases of increased thromboembolic events have been identified in patients administered IVIG products [1-3]; a specific brand of IVIG products was temporarily withdrawn from the market in 2010 owing to concerns over thromboembolic events [4, 5]. Because these issues persisted, in 2013 the U.S. Food and Drug Administration mandated that IVIG products carry warnings regarding potential thromboembolic events [6-8]. Thromboembolic events following IVIG administration are primarily caused by activated coagulation factor XI (FXIa) [8-10]. Consequently, several regulatory agencies have emphasized the need for processes that remove FXIa. Managing FXI levels in immunoglobin G (IgG) products is critical, as FXI can be converted into its activated form, FXIa [11-13]. FXIa is tightly regulated, as it serves as an immediate safety indicator directly linked to thrombogenic risk. However, quantification of the inactive precursor, FXI, also plays a critical role in terms of product efficacy and potential risk management. FXI represents a potential risk factor, as it can be gradually activated in vivo after administration, potentially leading to a delayed thrombotic response [28]. Therefore, continuous monitoring of total FXI levels is essential.

Several analytical methods are available for the determination of FXI concentration in IVIG products, including conversion assays, liquid chromatography – tandem mass spectrometry

(LC–MS/MS), western blotting, and sandwich enzyme-linked immunosorbent assay (ELISA) [27, 29, 30, 31]. The LC–MS/MS does not rely on antibodies and is therefore free from heterologous antibody interference; however, it requires expensive instrumentation and a high level of technical expertise. In contrast, western blotting has limited sensitivity for trace-level analysis (reference to be added). In contrast, sandwich ELISA offers exceptionally high sensitivity, enabling the detection of trace amounts of FXI, and allows simultaneous analysis of large numbers of samples, providing superior time and cost efficiency.

A sandwich ELISA, which is highly specific and sensitive, is generally used to evaluate the FXI content [13, 14]. It also allows for quantitative measurement of the target protein in the sample [15]. However, the choice of capture and detection antibodies in the assay can lead to non-specific reactions, which may increase background noise [15, 16]. Blocking agents are typically used to prevent interference from non-specific binding in ELISA. Bovine serum albumin (BSA) is commonly used as a blocking agent; however, its quality may vary among production processes and batches, which can lead to variability in the results, even under identical testing conditions. Therefore, the suitability of BSA in the assay must be screened to optimize its use [17].

Human immunoglobulins present in product are a potential source of interference in immunoassay analysis [18, 19]. These immunoglobulins include polyspecific antibodies with multiple binding specificities, rheumatoid factor–like autoantibodies, and antibodies generated as a result of exposure. Such interfering immunoglobulins are referred to as heterophilic antibodies and may cause assay interference in immunoassays, including sandwich ELISA [20, 21, 32]. Heterophilic antibodies can interfere with immunoassays, potentially disrupting the test and causing falsely elevated or reduced measured values. [22, 23]. These antibodies can bind to the Fc regions of both the capture and detection antibodies, causing cross-linking in the absence of the target analyte, leading to a false-positive result. In particular, sandwich ELISA is highly sensitive to interference from heterologous antibodies; therefore, minimizing the interference from heterophilic antibodies is crucial. Methods for blocking the interference from heterophilic antibodies include polyethylene glycol precipitation, precipitation using Protein L, the use of blocking agents, and direct treatment of samples with purified IgG [24-26].

IVIG products are pharmaceutical preparations in which highly purified human IgG is isolated from human plasma. While other antibodies and impurities are present at very low concentrations, human IgG is present at a high concentration. In the present study, we aimed to develop and validate a sandwich ELISA to measure FXI content, while minimizing interference from the high-concentration IgG in the samples. We hypothesized that purified human IgG (Fc) could act as a heterophilic antibody blocker, neutralizing non-specific interactions and thereby enhancing the reliability of the assay.

## Materials and Methods

### Materials

BSA (cat. A4737, A4612, A7638, A2153, A7030, and A3294) and sulfuric acid solution (1.09072) were purchased from Sigma-Aldrich (USA). Goat anti-human IgG (Fc) (L15406G) was purchased from Meridian Life Sciences (USA). An intermediate refers to a material obtained at a middle stage of the purification process, whereas the product is the final material obtained after the completion of purification. The samples were prepared in accordance with the same manufacturing process and quality standards as those used for commercial production. The samples were aliquoted and stored at –70°C and 4°C, respectively, until required.

### Measurement of FXI Using an ELISA

FXI content was measured using a Human Factor XI ELISA kit (Innovative Research, USA). The dilution buffer was prepared by adding a 0.3% IgG blocker(Goat anti-human IgG (Fc)) to 1x Tris-buffered saline (BioSesang, Republic of Korea) supplemented with BSA. Prepared standards and samples were loaded onto a plate coated with anti-factor XI antibody and incubated at 37°C for 30 min. After washing four times with wash buffer, the plate was treated with a biotin-labeled FXI antibody and incubated for 30 min at 37°C. After four washes, avidin-linked horseradish peroxidase was applied. Unbound reactants were removed by washing, and the plate was reacted with the 3,3′,5,5′-tetramethylbenzidine substrate solution to initiate a colorimetric reaction. The reaction was terminated by the addition of a stop solution, and measurements were obtained at wavelengths of 450 and 650 nm using a microplate reader (Spectramax Plus 384, Molecular devices, USA). The FXI content of the samples was analyzed using Softmax (Molecular devices). The standard curve was fitted using a four-parameter logistic model and utilized for calculating the sample values.

Different concentrations of the IgG blocker were used to confirm the inhibitory effect on human IgG interference following IgG blocker treatment. FXI was measured after adding 0.3%, 3.5%, and 10% IgG blocker to the sample dilution buffer.

### Assay Validation

The Validation of Analytical Procedures (Q2(R^2^)) included evaluating accuracy, precision, linearity, and limit of quantification in accordance with the ICH guidelines.

### Preparation of the FXI-Deficient Sample

To reproduce a condition in which FXI was selectively removed while maintaining an IgG matrix equivalent to that of the actual product, a commercially manufactured product was subjected to repeated execution of the primary FXI removal step within the manufacturing process to maximize FXI depletion. Two additional cation exchange (CEX) chromatography steps were performed to further remove residual FXI present in the product. Following the CEX process, the samples were concentrated through the ultrafiltration/diafiltration (UF/DF) step to achieve an IgG concentration equivalent to that of the final product. This sample was defined as FXI-deficient and was used to identify false-positive signals during FXI content measurement.

### Statistical Analysis

The data were analyzed using GraphPad Prism 10 (GraphPad, USA) to evaluate the relevance of the test outcomes. To assess the equivalence regarding the presence or absence of IgG blocker treatment, two-way analysis of variance was performed. A *p*-value of 0.05 or greater indicated absence of a significant difference.

## Results

### Confirmation of FXI Content in Samples

The ELISA assay conditions were established according to the manufacturer’s instructions for the commercially available kit. After optimization, the blank optical density (OD) value was < 0.1, indicating minimal non-specific reactions. However, further analysis revealed that discrepancies in measured FXI content were influenced by the BSA product and lot (Table 1). The sample consisted of highly concentrated immunoglobulins obtained from purified human plasma, containing a mixture of various human immunoglobulin subclasses. In sandwich ELISA, high levels of IgG can act as heterophilic antibodies, potentially causing false-positive or -negative signals. This mechanism also caused this phenomenon.

### Effect of IgG Blocker on FXI Analysis

**Fig. 1:**
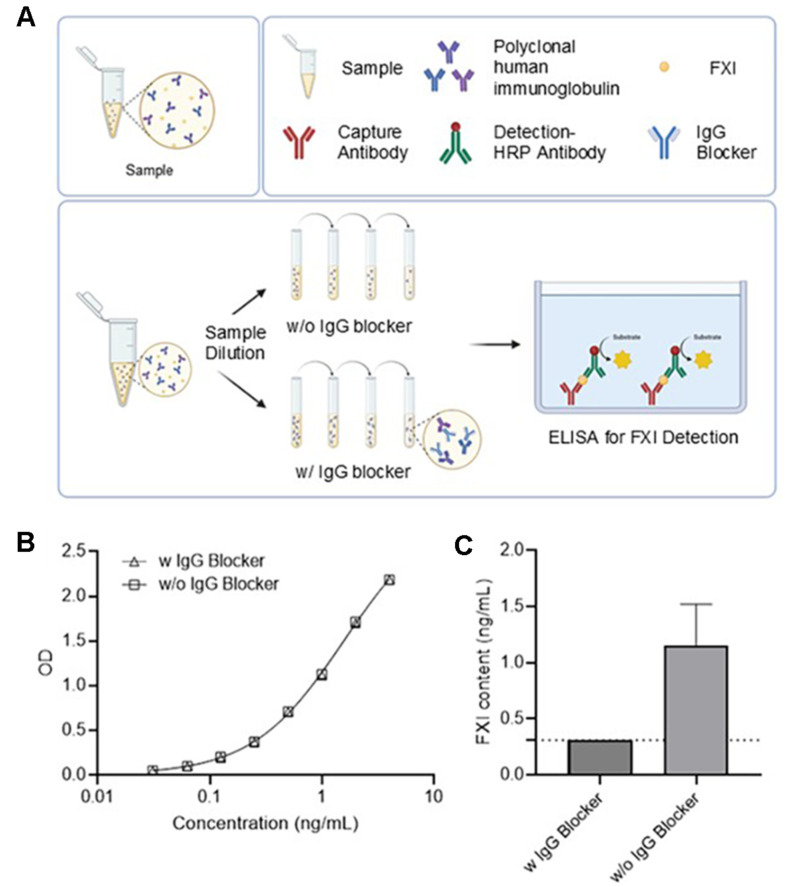
(**A**) Schemes of FXI ELISA steps. A sandwich ELISA was used to measure FXI content. All procedures were performed identically, except for the inclusion or exclusion of the IgG blocker treatment in the sample dilution buffer. (**B**) Absorbance measurements of the standard curve with and without IgG blocker treatment. The standard curve was fitted using a four-parameter logistic curve and measured within a range of 0.031–4 ng/ml. The limit of quantification was identical at 0.031 ng/mL regardless of the addition of the IgG blocker. Under both conditions, the coefficient of determination (R^2^) of the standard curve was confirmed to be 0.999. (**C**) Mean and SD of FXI content (*n* = 3). The dashed line indicates the concentration calculated as the lower limit of detection multiplied by the dilution factor (< 0.31 ng/ml). FXI, Factor XI; SD, standard deviation; IgG, immunoglobulin G; ELISA, enzyme-linked immunosorbent assay.

An IgG blocker was used to confirm whether IgG in the sample acted as a heterophilic antibody (Fig. 1A). The IgG blocker was applied to the sample and purified FXI standard to evaluate its effect (Fig. 1B and 1C). No difference in the OD or R^2^ values with or without blocker treatment was observed for the purified FXI standard. In the samples, changes in FXI content were observed depending on the presence or absence of the IgG blocker. FXI in samples without IgG blocker treatment was detected at 0.93–1.58 ng/ml. Conversely, FXI was not detected when the samples were treated with an IgG blocker. This finding suggests that IgG in the samples could interfere and cause false-positive signals, and that this interference was eliminated by treatment with the IgG blocker.

### Interference Inhibition Effect by IgG Blocker

**Fig. 2:**
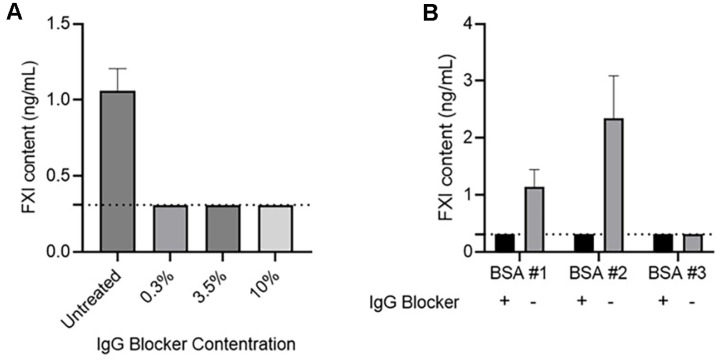
Effect of IgG matrix inhibition. (**A**) FXI content was analyzed after adding 0.3%, 3.5%, and 10% buffer-diluted IgG blocker solutions to determine the effect of IgG blocker concentration. The mean and SDs of the measured FXI content (*n* = 3) are shown. The dashed line indicates the concentration calculated as the lower limit of detection multiplied by the dilution factor (< 0.31 ng/ml). (**B**) Dilution buffers were prepared using three types of bovine serum albumin. FXI content was then evaluated with and without IgG blocker addition. Results represent the mean and SD of measurements within the sample (*n* = 3). The dashed line indicates the concentration calculated as the lower limit of detection multiplied by the dilution factor (< 0.31 ng/ml). FXI, Factor XI; SD, standard deviation; IgG, immunoglobulin G.

Three different concentrations of IgG blocker were applied to examine the effects of the IgG blocker. The FXI concentration in the samples ranged from 0.97 to 1.23 ng/mL. FXI was not detected when an IgG blocker was added to the same sample (Fig. 2A). The minimum concentration tested (0.3%) represented a small amount, equivalent to 0.03% in the diluted sample. This result confirmed that even a small amount could reduce the interference caused by IgG in the sample.

The IgG interference inhibition of the IgG blocker was evaluated using three types of BSA that showed differences in FXI content (Fig. 2B). For BSA #1 and #2, FXI levels were detected at 0.88–1.47 ng/ml and 1.49–2.72 ng/ml, respectively, without IgG blocker treatment. In contrast, FXI was not detected when an IgG blocker was applied. No difference was observed in BSA #3 before and after IgG blocker treatment. These results confirm that false-positive can occur because of the sample matrix, and that IgG blocker treatment eliminates the interference caused by IgG in the sample.

### Confirmation of False-Positive Signals in FXI-Deficient Samples

**Fig. 3:**
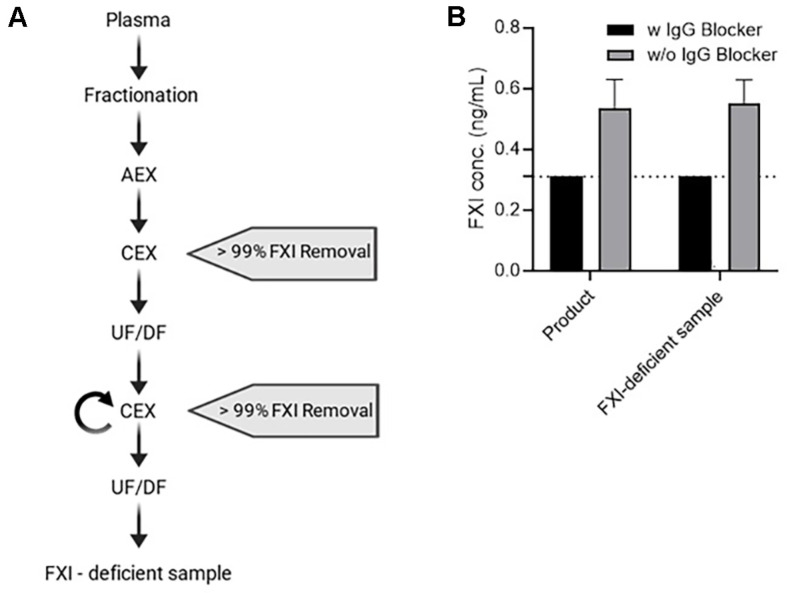
FXI content results for FXI-deficient samples. (**A**) Schematic of the FXI-deficient sample preparation process. Pooled human plasma was used as the starting material, and the product was manufactured through a high purity IgG production process based on cold ethanol fractionation. The manufacturing process consisted of ethanol fractionation, anion exchange (AEX) chromatography, CEX, and UF/DF steps. After two rounds of CEX, the sample underwent UF/DF to achieve a uniform IgG concentration. IgG content was analyzed and confirmed using a nephelometer (data not shown). (**B**) The product and the FXI-deficient sample were analyzed in the same assay system. The result represents the mean and SD of the measured values in the sample (*n* = 3). The dashed line indicates the concentration calculated as the lower limit of detection multiplied by the dilution factor (< 0.31 ng/ml). FXI, Factor XI; SD, standard deviation; IgG, immunoglobulin G.

FXI-deficient samples were prepared to confirm the false positives caused by IgG. The CEX process used for sample preparation removed > 99% of the FXI [27]. After repeating this process twice, UF/DF was performed to ensure that the samples contained high concentrations of IgG (Fig. 3A). The FXI levels in the sample and FXI-deficient samples without IgG blocker treatment were measured at 0.43–0.59 ng/ml and 0.51–0.64 ng/ml, respectively. Even in samples from which FXI was removed, detectable levels of FXI were observed. Conversely, FXI was not detected in any of the samples treated with the IgG blocker (Fig. 3B). Therefore, FXI detected in the samples was confirmed to be a false-positive reaction, and the IgG blocker effectively reduced IgG interference.

### Effect of IgG Concentration on FXI Content Measurement

**Fig. 4:**
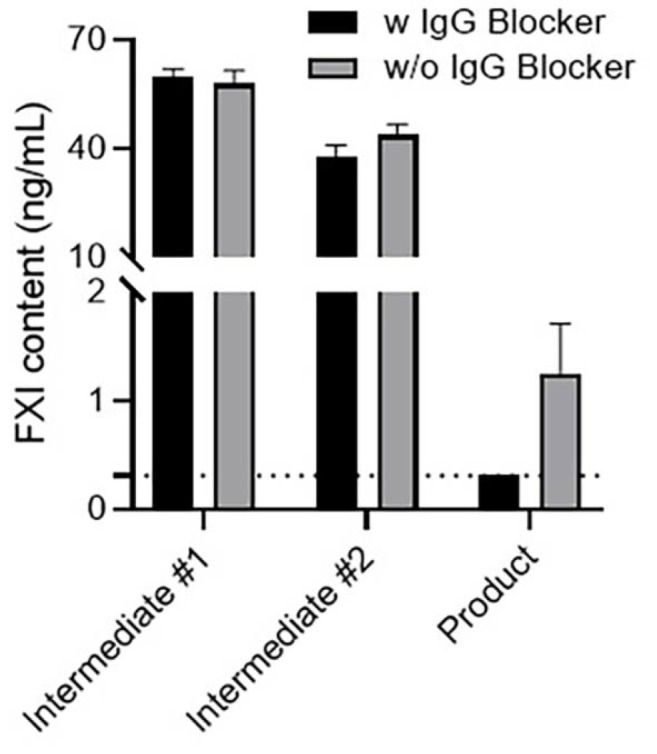
FXI content in samples with different IgG levels following IgG blocker treatment. The FXI content was evaluated in three samples (two intermediates and one product) with different IgG concentrations, with and without IgG blocker treatment. The IgG level in the product with a high IgG concentration was 100.2 mg/ml, whereas the IgG levels in Intermediate #1 and #2 were 2.4 and 4.4 mg/ml, respectively. Three samples were analyzed using an IgG blocker to examine FXI. The results are presented as the mean and SD of the measured values in the sample (*n* = 2). The dashed line indicates the concentration calculated as the lower limit of detection multiplied by the dilution factor (< 0.31 ng/ml). FXI, Factor XI; SD, standard deviation; IgG, immunoglobulin G.

During the IgG purification process, samples with low IgG content were obtained to compare the interference effects based on IgG concentration (Fig. 4). Depending on the IgG blocker treatment conditions, the FXI levels for Intermediate #1 and #2 were similar at 55.50–58.44 ng/ml and 40.10–45.90 ng/ml, respectively. In contrast, the sample containing high-concentration IgG was detected at 1.47–1.58 ng/ml without blocker treatment, but not after blocker treatment. Because IgG interference was observed only in samples with high IgG concentrations, we conclude that it is induced by these high IgG concentrations within the sample, potentially leading to false-positive results.

### Equivalence Assessment before and after IgG Blocker Treatment

**Fig. 5:**
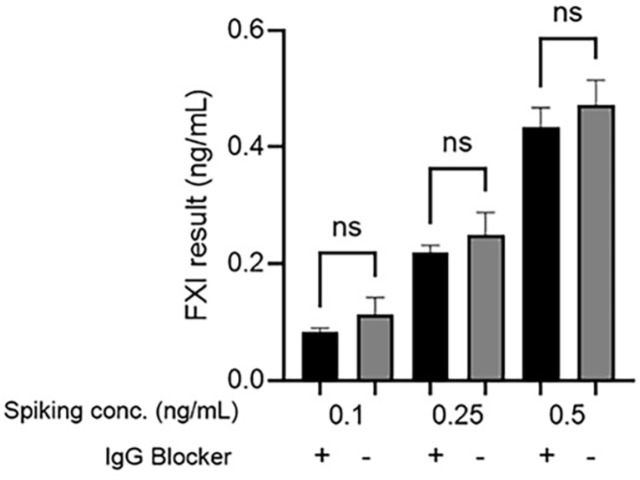
FXI results with and without IgG blocker treatment. FXI was spiked into samples at concentrations of 0.1, 0.25, and 0.5 ng/ml and analyzed using ELISA. All data are presented as the mean and SD (*n* = 5). A two-way ANOVA was performed using the GraphPad Prism 10.6.0. Differences were considered insignificant when the *p*-value was greater than or equal to 0.05. ns: not significant. FXI, Factor XI; SD, standard deviation; IgG, immunoglobulin G; ELISA, enzymelinked immunosorbent assay; ANOVA, analysis of variance.

FXI at concentrations of 0.1, 0.25, and 0.5 ng/ml was spiked into the samples to assess equivalence before and after IgG blocker treatment. At each spiking concentration, FXI levels were comparable with and without IgG blocker treatment (Fig. 5). Statistical analysis showed *p*-values of 0.3264, 0.2894, and 0.1642 at each spiking concentration, confirming no significant difference in the results based on IgG blocker treatment conditions.

### Assay Validation

**Precision and accuracy analysis.** The assay precision and accuracy were evaluated (Table 2). Sample precision was confirmed by performing three replicate tests, with a coefficient of variation (CV, %) of 9.1% each, thereby satisfying the acceptance criterion of ≤ 20%. The FXI standards at low (0.031 ng/ml), medium (0.25 ng/ml), and high (2 ng/ml) concentrations were spiked into the samples to assess the accuracy of the results. The spiked recovery rates for all concentrations met the acceptance criteria of 70%–130%.

**Fig. 6:**
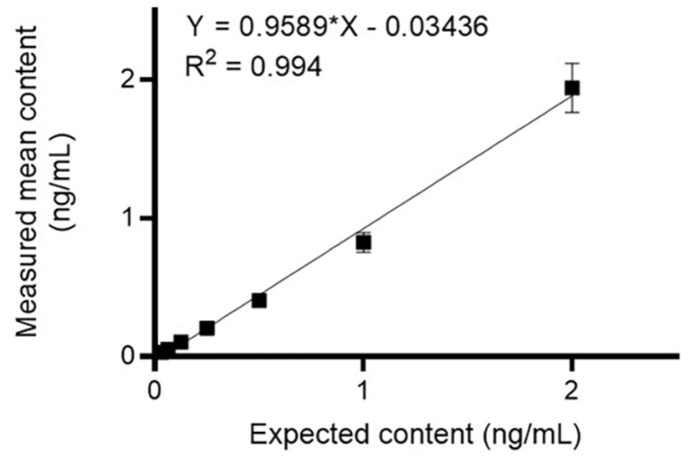
Linearity evaluation. Linearity was evaluated by spiking seven concentrations of FXI into the product. Using the theoretical value of the spiked sample X and the measured value as Y, the linear regression equation, coefficient of determination (R^2^), slope, and y-intercept between log X and log Y were derived using the GraphPad Prism software. Dots represent the mean and SD of the measured values at each concentration (*n* = 3). FXI, Factor XI; SD, standard deviation.

**Linearity and limit of quantification analysis.** Linearity was evaluated by spiking seven concentrations of FXI (0.0625, 0.125, 0.25, 0.5, 1, 2, and 4 ng/ml) into the samples (Fig. 6). The CV (%) at each concentration level reached 13.7%, while the relative error fluctuated between –25.8% and 2.4%, thereby confirming its suitability within the established criteria. The assay range was 0.031–2 ng/ml, and the limit of quantification was 0.031 ng/ml, representing the minimum concentration at which acceptable precision and accuracy were achieved.

## Discussion

The samples used in this study had previously been evaluated for FXI clearance and underwent CEX chromatography to remove FXI. Although residual FXI was detected in the intermediate during processing, its level fell below the limit of quantification after CEX step. Overall, the CEX process reduced FXI content by more than 99%, indicating that only minimal levels of FXI remained in the final sample [27]. Furthermore, the residual FXI content in the samples was evaluated using sandwich ELISA. Despite the use of BSA as a blocking agent, non-specific reactions were observed in the FXI measurements of the research batch samples, depending on the type of BSA. The blank OD value was significantly lower (below 0.1), confirming the blocking effect of BSA. Subsequent studies also failed to demonstrate sufficient efficacy in suppressing non-specific reactions at higher concentrations of BSA (data not shown). However, interference from high concentrations of IgG was observed, indicating that further research on other interference factors was necessary. Considering these findings, it is evident that although BSA adequately blocks non-specific reactions in sandwich ELISA, a method to mitigate the interference effect of IgG in the sample is necessary.

The effect of IgG-blocker treatment was discernible exclusively in specific samples characterized by elevated IgG concentrations, with no discernible signal recorded in the measured values following application of the IgG-blocker. The interference observed within the sample was attributed to elevated concentrations of IgG, and the subsequent application of an IgG blocker indicated that the interference within the sample was mitigated. Without applying the IgG blocker, the measured values varied depending on the type of BSA. Conversely, the application of an IgG blocker eliminated interference within the sample, irrespective of the BSA type. These findings suggest the necessity of interference elimination strategies to prevent IgG interference within the sample. To demonstrate that IgG in the sample is an interference factor, causing false-positive signals, samples with varying IgG concentrations were tested, and the FXI was compared and evaluated. In the intermediate sample, which was characterized by a low IgG concentration, no significant disparities in FXI were observed between the treatment and control groups.

In contrast, in the sample with a high IgG concentration, a difference in FXI content was observed following IgG blocker treatment. The effect of FXI on IgG blocker treatment was observed only in samples with high IgG concentrations, suggesting that high IgG concentrations cause false-positive FXI signals. We hypothesized that if FXI content was measured in FXI-deficient samples, high-concentration IgG would act as a heterophilic antibody, inducing a false-positive signal. FXI was detected in both the sample and FXI-deficient samples in the absence of an IgG blocker, whereas no FXI was detected when the IgG blocker was present. Consequently, the confirmation that FXI was not detected until after IgG blocker treatment demonstrated that the false-positive result was due to the high concentration of IgG in the sample.

The IgG blocker is an antibody-based product with a high unit cost; however, only a small amount is required to achieve effective interference reduction, making it advantageous for improving assay accuracy.

Furthermore, the precision, accuracy, linearity, and limit of quantification of ELISA using the IgG blocker were validated. The results met the acceptable criteria, thus proving that the assay was both accurate and reliable by eliminating interference from high concentrations of IgG in the sample.

In conclusion, in sandwich ELISA-based measurements of samples containing high concentrations of IgG, the potential of IgG to act as a heterophilic antibody, resulting in non-specific reactions, should be considered. Consequently, false-positive values can be generated, which makes it crucial to eliminate interference from heterophilic antibodies during sandwich ELISA. Despite the existence of numerous methodologies for eliminating this interference, the present study used an IgG blocker to eradicate it. This approach enhanced the reliability and precision of the assay by providing accurate results for the evaluation of impurities in IVIG products. The developed ELISA test accurately assessed FXI, regardless of the IgG content in the sample. These conditions are expected to be broadly applicable to quantitative FXI assays for assessing impurities in high-purity IgG products.

## Figures and Tables

**Table 1:** Factor XI concentrations in samples based on bovine serum albumin products.

BSA		FXI concentration (ng/ml)
Cat. No.	Lot No.	
A4737	SLCC4406	< 0.31
	SCLK2817	0.93
	SLCG9275	1.58
A7638	SLCL1838	2.72
A4612	SLBM2472V	< 0.31
A2153	SLCB9433	< 0.31
	SLCD0987	0.35
	SLCC9421	0.45
A7030	SLCD0756	1.46
	SLCC6453	0.38
A3294	SLCD6757	0.43

BSA, bovine serum albumin; FXI, factor XI

**Table 2:** Results for assay precision and accuracy.

Run	Precision	Accuracy		
	FXI concentration (ng/ml)	Recovery of spiked FXI (%)		
		Low	Medium	High
1	2.035	112.7	87.6	101.7
2	1.735	93.6	75.0	86.7
3	2.049	86.5	84.1	102.4
Average (ng/ml)	1.939	N/A		
CV (%)	9.1	N/A		

The coefficient of variation (CV, %) was calculated using the spiked sample results for precision because the FXI content in all samples was below the minimum concentration of the standard. Intermediate precision was determined using a single operator performing three replicates. Spiked recovery (%) was calculated using the following formula: Spiked recovery (%) = (Spiked FXI result – FXI result) ÷ Spiked concentration × 100
